# Gender and Age Differences in ADHD Symptoms and Co-occurring Depression and Anxiety Symptoms Among Children and Adolescents in the BELLA Study

**DOI:** 10.1007/s10578-023-01622-w

**Published:** 2023-10-18

**Authors:** Martha Gilbert, Maren Boecker, Franziska Reiss, Anne Kaman, Michael Erhart, Robert Schlack, Joachim Westenhöfer, Manfred Döpfner, Ulrike Ravens-Sieberer

**Affiliations:** 1 Department of Child and Adolescent Psychiatry, Psychotherapy, and Psychosomatics, University Medical Center Hamburg-Eppendorf, Hamburg, Germany; 2 Alice Salomon University of Applied Sciences, Berlin, Germany; 3 Apollon University of Applied Sciences, Bremen, Germany; 4 Department of Epidemiology and Health Monitoring, Robert Koch Institute, Berlin, Germany; 5 Department of Health Sciences, Faculty of Life Sciences, Competence Center Health, Hamburg University of Applied Sciences, Hamburg, Germany; 6 Department of Child and Adolescent Psychiatry, Psychosomatics and Psychotherapy, Faculty of Medicine, University Hospital Cologne, University of Cologne, Cologne, Germany; 7 Department of Child and Adolescent Psychiatry, Psychotherapy, and Psychosomatics, Research Section “Child Public Health”, University Medical Center Hamburg-Eppendorf, Martinistraße 52, W 29, 20246 Hamburg, Germany

**Keywords:** ADHD, Gender differences, Mental health, Adolescents, Depression, Anxiety

## Abstract

Attention-deficit/hyperactivity disorder (ADHD) is one of the most diagnosed neurodevelopmental disorders of childhood. Current studies addressing gender and age differences in ADHD are lacking. The present study aims to fill this research gap by dimensionally evaluating gender and age differences in ADHD symptoms, as measured by a DSM-5-based parent rating scale, in children and adolescents who participated in the two-year follow-up of the community-based BELLA study (*n* = 1326). Associations between ADHD symptoms and depression symptoms and anxiety symptoms were also examined. Multiple linear regressions revealed significant associations between gender and all ADHD symptoms. Age was significantly associated with hyperactive/impulsive symptoms. Additional multiple linear regressions demonstrated significant positive associations between depression and anxiety symptoms and ADHD symptoms. Further, female gender was found to be positively associated with both depression and anxiety symptoms. These findings may suggest a need for more gender-specific approaches to ADHD diagnosis and treatment, as well as more research into the intersections of ADHD and depression and anxiety symptoms in children and adolescents.

## Background

Attention-deficit/hyperactivity disorder (ADHD) is characterized by signs of developmentally inappropriate inattentiveness, impulsivity, and/or hyperactivity. According to the DSM-5 and ICD-10, these symptoms must occur over a period of at least six months and must have a negative impact on academic, occupational, and social functioning [1–4]. Children and adolescents with ADHD often struggle with learning disabilities, mood disorders and relationships with peers which can lead to reduced subjective health-related quality of life [5] and comorbidities like depression and anxiety [6]. Further, recent studies demonstrate that the majority of children diagnosed with ADHD continue to experience symptoms of ADHD into adulthood [7–9].

Prevalence rates of ADHD vary greatly depending on study methods and design, but a meta-analysis of studies of mental disorders among children and adolescents by Polanczyk et al. [10] calculated a worldwide prevalence of 3.4% for ADHD. This prevalence rate is significantly lower than the pooled prevalence of 5.3% that [11] estimated eight years earlier in a meta-analysis which looked only at ADHD studies. This discrepancy could indicate the sensitivity of ADHD prevalence estimates to varying assessment methods (e.g. clinical interview versus assessment instrument) and varying samples (e.g. clinical versus community samples) and underscores the need for more studies of this nature and also clear methodological guidelines and practices for prevalence calculation [10]. However, changes in prevalences could also be related to diagnosis trends or individual changes over the lifespan of a child or adolescent diagnosed with ADHD. More in-depth analysis of age differences in ADHD presentation could help identify patterns and inform clinical practices and diagnostic approaches.

The rate of ADHD diagnosis also varies greatly depending on gender. A meta-analysis of ADHD studies by Willcutt [12] found that males were more likely than females to meet criteria for an overall diagnosis of ADHD and for each of the DSM-IV subtypes. Furthermore, DSM-IV criteria for ADHD were created primarily based on observations of males [13], and in DSM-5 field studies males represented a greater percentage of the sample than females [14]. These factors may help indicate why DSM criteria are often more sensitive to boys than girls in terms of ADHD symptoms. Another reason for a potential underdiagnosis of ADHD in girls could be the fact that a larger proportion of girls than boys present with inattentive-type ADHD [12] making it less likely that their behavior is conspicuous and perceived as disruptive. Girls who are diagnosed with ADHD rate higher on internalizing symptoms and are more likely to struggle with comorbid conditions such as depression and anxiety [15].

Further, girls’ inattentive behavior may look like: being disorganized, overwhelmed, easily distracted or lacking in effort and motivation [16]. These symptoms may not be recognized as ADHD symptoms or may be ignored because of their apparent mildness. Several studies have found that parents and teachers are less bothered by and see “feminine” ADHD diagnostic items as less problematic than “masculine” ones [17, 18]. Girls may also display adaptive or compliant behavior or develop coping strategies that mask their symptoms [19, 20]. Further, a study by Mowlem et al. [19] found that parents under rated diagnosed girls’ hyperactive/impulsive symptoms compared to the more objective *Parental Account of Childhood Symptoms*, an investigator-rated semi-structured interview, that they also completed. At the same time, parents had a tendency to over report hyperactive and impulsive behavior in boys. This could indicate that gender-specific biases in perceptions of child behavior exist. Girls with ADHD may also be overlooked or their symptoms may be misinterpreted if they are able to compensate for their difficulties, receive a high level of support, or live in a highly structured environment [16]. Additionally, new evidence suggests that, in both clinical and community samples, over the course of adolescence and adulthood, the proportion of female ADHD cases increases, while the proportion of male cases decreases. This could suggest that gender differences in ADHD symptoms lessen with age [21].

ADHD symptoms have been shown to decrease with age. In their meta-analysis, Willcutt et al. (2012) found that the estimated prevalence of ADHD hyperactive-impulsive type was highest in preschool children, but then declined steadily over the course of childhood into adolescence. These findings illustrate the potential evolution of ADHD symptoms, wherein hyperactive and impulsive symptoms are replaced by more inattentive symptoms as the individual ages. In a large, representative sample, Ramtekkar et al. [22] found that although ADHD symptom counts were lower at older ages, older individuals with a lifetime ADHD diagnosis according to DSM-IV had markedly higher current ADHD symptoms when compared to the total sample. Conversely, anxiety and depression symptoms have been found to be more common among older children and adolescents [23].

Co-occurring mental health problems with an ADHD diagnosis are frequent and can sometimes exacerbate the symptoms of each individual disorder. Depression and anxiety are two of the most frequently co-occurring mental health problems with ADHD [24]. In their meta-analysis, Meinzer et al. [25] found that in 88.2% of their reviewed cross-sectional studies children and adolescents with a diagnosis of ADHD had a significantly higher rate of depression relative to those without ADHD.

As some children and adolescents, especially girls, with impairing ADHD symptoms do not meet the clinical threshold for an ADHD diagnosis [26], it can be useful to evaluate ADHD dimensionally, instead of categorically, where a minimum of symptoms for each dimension of ADHD must be met. This dimensional approach also can serve to identify patterns among ADHD symptoms in community samples, where likely most of the subjects have not received an ADHD diagnosis. Evaluation of ADHD on a dimensional, symptom-level can also help to assess trends without relying on prevalence rates. Furthermore, closer inspection of gender and age difference in the subscales Inattention and Hyperactivity/Impulsivity could provide valuable insight for clinicians, as well as future directions for research.

The objective of the present study was to examine gender and age differences in parent-reported child and adolescent ADHD symptoms using data from a large population-based sample in Germany. Moreover, we wanted to investigate whether higher levels of ADHD symptoms would predict higher levels of depression and anxiety symptoms.

This study aims to contribute to existing literature on the ways in which ADHD manifests differently in girls than in boys as well as in older versus younger children. It was hypothesized that girls and older adolescents, as well as older girls, would be more likely to present with lower ADHD symptom scores using the parent-reported FBB-ADHS. Girls were anticipated to present with higher scores on the Inattention subscale, when compared to the Hyperactivity/Impulsivity subscale, and younger children were expected to score higher on the Hyperactivity/Impulsivity subscale. Building on existing research, it was predicted that rates of depression and anxiety symptoms would be higher among children and adolescents presenting higher rates of ADHD symptoms, as well as for girls and older adolescents.

Findings from the study could add to the body of evidence that suggests that there could be gender differences in the efficacy of diagnostic tools for assessing ADHD and that gender-specific approaches need to be implemented to meet the unique needs of girls and women with ADHD. Associations between ADHD and depression and anxiety symptoms help to highlight the necessity of considering co-occurring diagnoses in interaction with one another and not merely independently. The calculation of effect sizes can help to contextualize the magnitude of these differences and thus their significance for future research directions.

## Methods

### Study Design and Sample

The study sample is a subsample of the longitudinal BELLA study (*Study of Behaviour and Wellbeing of Children and Adolescents in Germany*), which is the mental health module of the *German National Health Interview and Examination Survey for Children and Adolescents* (KiGGS), conducted by the Robert Koch Institute. The BELLA study gathers data on mental health, health-related quality of life, and mental health care use, as well as on risk and protective factors for mental health problems for German children, adolescents, and young adults. The baseline sample of the BELLA study consisted of a representative subsample of KiGGS including *n* = 2863 families with children aged 7–17. For all measurement points of the BELLA study, approval from the ethics committee of the University Hospital Charité in Berlin and the Federal Commissioner for Data Protection in Germany was obtained. Detailed information on the design, methods and results of the BELLA study can be found elsewhere [27]. For the current analyses, only data from the two-year follow-up of the BELLA study (2005–2008) was used. Participants who were 9 to 17 years of age at the two-year follow-up and provided complete data for ADHD, depression and anxiety symptoms were included. This resulted in a sample of 1326 participants.

### Measures

In the BELLA study, existing well-established, standardized instruments were administered. To ensure comparability of results across age groups, only parent-reported data was used for these analyses, as these were available for all participants.

#### Sociodemographics

Information on the children and adolescents’ gender and age, as well as the families’ socioeconomic status, and migration background was collected. Indication of socioeconomic status was determined based on parental education employing the Comparative Analysis of Social Mobility in Industrial Nations (CASMIN) classification [28]. Parents of participants were identified as having achieved low, medium, or high education. Migration background was assessed following Schenk [29]. Participants were categorized as having a migration background if they had immigrated to Germany and had at least one parent born in a country other than Germany or if both parents immigrated to Germany or did not hold German citizenship.

#### ADHD Symptoms

The parent-reported German ADHD Rating Scale (FBB-ADHS) [30] was administered to assess ADHD symptoms. This scale consists of 20 items reflecting the symptom criteria from both the ICD-10 and DSM-5. Parents indicated the severity of ADHD symptoms on a 4-point answer scale ranging from “not true” (0) to “especially true” (3). Using the FBB-ADHS, a Total Scale Score can be calculated using all items of the instrument, with higher scores indicating a higher degree of inattentive or hyperactive/impulsive symptoms. The subscales Inattention, consisting of 9 items reflecting internalizing symptoms of ADHD, and Hyperactivity/Impulsivity, consisting of 11 items indicating externalizing symptoms of ADHD, can also be calculated individually. For the complete scale, as well as for the two subscales, the scale scores range from 0 to 3 and represent the mean of the respective individual item scores. Erhart and colleagues [31] determined that the FBB-ADHS demonstrated reliability and good to excellent internal consistency (Cronbach’s α = 0.73−0.90) as well as factorial validity (RMSEA = 0.06).

#### Depression Symptoms

To assess depression symptoms, the parent-reported German version of the Center for Epidemiological Studies Depression Scale for Children (CES-DC) [32, 33] was used. It includes 20 items that assess positive affect, as well as cognitive, behavioral, affective, and somatic symptoms associated with depression over the last week. Each item can be rated on a 4-point Likert scale ranging from “not at all” (0) to “a lot” (3). The sum scale score ranges from 0 to 60, with higher scores indicating more depression symptoms. The CES-DC has shown good factorial validity and stability across age and informant versions [32].

#### Anxiety Symptoms

Anxiety was assessed using the parent-reported German version of the SCARED questionnaire (Screen for Child Anxiety Related Emotional Disorders) [34, [35]. This questionnaire contains 41 items, which can be assigned to five subscales according to the factors of the instrument: panic/somatic, generalized anxiety, separation anxiety, social phobia, and school phobia. Items could be answered on a 3-point Likert scale ranging from “not true” (0) to “very true” (2). A scale sum score was calculated with values ranging from 0 to 82. A higher score indicates more anxiety. The SCARED questionnaire has demonstrated good internal consistency and discriminant validity [34].

### Data Analysis

For these analyses, only participants with no missing data on the scales FBB-ADHS, CES-DC, and SCARED were used. This procedure has been followed in several other publications of the BELLA study [27, 36, 37]. Before conducting the statistical analyses, an a priori power calculation was conducted using G*Power 3.1 [38] to assess the necessary minimum sample size for multiple linear regression. The minimum sample size based on a statistical significance of *p* (α) < 0.05 and a power of *p* (1ß) = 0.8 for a small-medium effect as measured by *f* ² = 0.08 was n = 187.

#### ADHD Symptoms

Mean sum scores and standard deviations for the Total Score as well as for the subscales Inattention and Hyperactivity/Impulsivity were calculated for boys and girls, and also the age groups: 9–10, 11–13, and 14–17. These age groups mirror those used for clinical cut-offs in the DISYPS-III. To demonstrate the distribution of the scores, quartiles for all three scales were also calculated. Further, the effect size Cohen’s *d* was calculated for the mean sum scores for all three scales by gender, wherein 0.2 indicates a small effect size, 0.5 a moderate effect size, and 0.8 or higher a large effect size [39].

#### ADHD Symptoms and Co-occurring Depression and Anxiety Symptoms

Associations between age, gender, and ADHD symptoms, as measured by the FBB-ADHS Total Score and two subscales Inattention and Hyperactivity/Impulsivity, were performed with three linear regression models. An interaction term of age × gender was created to examine the effects of age and gender on ADHD symptoms and added to the models. To assess effects of ADHD symptoms, gender, and age on depression and anxiety symptoms, two multiple linear regression models were performed. For all models, bivariate analyses, including Pearson and Spearman correlations and chi-square tests were conducted to examine potential associations between the variables. Correlation coefficients (r) between 0.1 and 0.3 were considered weak correlations, coefficients between 0.3 and 0.5 medium, and coefficients above 0.5 as evidence of strong correlations. The Variance Inflation Factor (VIF) was used to control for multicollinearity among the predictor variables. All predictor and control variables including: level of ADHD symptoms (for depression and anxiety regression models), age, gender, parental education, and migration background were entered simultaneously into the regression models. Effect sizes are reported as adjusted *R*², where 0.02 indicates a small effect size, 0.13 a medium effect size, and 0.26 or higher a large effect size [39]. Age was centered around the mean for the regression analyses. All analyses were performed with SPSS Version 27.

## Results

### Sample Characteristics

The analyzed sample consisted of n = 1326 children and adolescents aged 9–17 years (52.5% male). The mean age was 12.69, with a standard deviation of 2.55 (Mdn. = 12(10 to 15)). Most participants did not have a migration background (94%) and came from a household of medium (62.5%) or high (27.6%) parental education. A detailed description of the sample can be found in Table 1.

**Table 1:** Sociodemographic characteristics of the BELLA sample at two-year follow-up.

	BELLA sample (*n* = 1326)	
	*n* (%)	*M (SD)*
Age		12.69 (2.55)
9 to 10 years	340 (25.6)	
11 to 13 years	462 (34.8)	
14 to 17 years	524 (39.5)	
Gender		
Male	696 (52.5)	
Female	630 (45.5)	
Migration background		
No	1247 (94.0)	
Yes	77 (5.8)	
No information	2 (0.2)	
Parental education		
Low	127 (9.6)	
Medium	829 (62.5)	
High	366 (27.6)	
No information	4 (0.3)	

*M *mean, *SD*  standard deviation

### ADHD Symptoms

#### Mean Scores by Gender and Age Group

Overall, mean values on the Total Score and both subscales were relatively low, ranging between 0 (“never or rarely”) and 1 (“somewhat true”) in this representative sample of children and adolescents. On all scales, boys and the youngest age group (9–10) reported the highest mean scores. For both boys and girls, the highest mean scores were on the Inattention subscale, with values of 0.74 (*SD* = 0.53) and 0.56 (*SD* = 0.43) respectively. The lowest mean scores were found on the Hyperactivity/Impulsivity subscale for girls with a score of 0.29 (*SD* = 0.32) and the 14-17-year-olds with a score of 0.29 (*SD* = 0.37). For all mean scores and standard deviations as well as quartiles by gender and age group, see Table 2. Cohen’s *d* indicated a small effect of gender on the Total Score (*d* = 0.32), a small effect of gender on the Inattention subscale (*d* = 0.37), and no significant effect of gender on the Hyperactive/Impulsive subscale (*d* = 0.19).

**Table 2:** Mean scores FBB-ADHS total score and subscales by gender and age group

		Gender		Age group		
		Male	Female	9-10	11–13	14–17
FBB-ADHS Total Score						
Mean (SD)		0.53 (0.42)	0.41 (0.33)	0.51 (0.41)	0.48 (0.38)	0.44 (0.36)
Quartiles	25	0.25	0.20	0.25	0.24	0.20
	50	0.45	0.35	0.45	0.40	0.35
	75	0.70	0.55	0.65	0.61	0.60
FBB-ADHS Subscale Inattention						
Mean (SD)		0.74 (0.53)	0.56 (0.43)	0.69 (0.51)	0.66 (0.49)	0.62 (0.48)
Quartiles	25	0.33	0.22	0.33	0.33	0.22
	50	0.67	0.56	0.56	0.56	0.56
	75	1.00	0.78	0.89	0.89	0.89
FBB-ADHS Subscale Hyperactivity/Impulsivity						
Mean (SD)		0.36 (0.41)	0.29 (0.32)	0.37 (0.41)	0.34 (0.37)	0.29 (0.34)
Quartiles	25	0.09	0.09	0.09	0.09	0.09
	50	0.27	0.18	0.27	0.18	0.18
	75	0.55	0.36	0.54	0.45	0.36

*n* = 1326

#### Bivariate Analysis

The correlations between the different predictor variables and the dependent variables of the regression analyses can be found in Table 3. Only weak associations were found among sociodemographic variables, except for migration background and parental education (*r* = 0.072, *p* < 0.01). Total ADHD symptoms were significantly correlated with gender (*r* = − 0.159, *p* < 0.01), age (*r* = 0.086, *p* < 0.01), and parental education (*r* = − 0.123, *p* < 0.01), even though the correlation coefficients were of small size. Inattentive and hyperactive/impulsive ADHD symptoms also correlated with gender, age, and parental education. Depression symptoms (*r* = 0.382, *p* < 0.01) and anxiety symptoms (*r* = 0.369, *p* < 0.01) both strongly correlated with total ADHD symptoms.

**Table 3:** Zero-order correlations among variables

Variables	1	2	3	4	5	6	7	8
1. Gender ^a^	–							
2. Age ^c^	− 0.019	–						
3. Parental education ^b^	− 0.023	− 0.007	–					
4. Migration background ^a^	0.138	0.003	0.072**	–				
5. Total ADHD symptoms ^c^	− 0.159**	− 0.086**	− 0.123**	0.025	–			
6. Inattentive ADHD symptoms ^c^	− 0.181**	− 0.061*	− 0.108**	0.053	0.911**	–		
7. Hyperactive/Impulsive ADHD symptoms ^c^	− 0.103**	− 0.095**	− 0.144**	− 0.010	0.895**	0.631**	–	
8. Depression symptoms ^c^	0.018	0.030	− 0.068*	− 0.010	0.382**	0.376**	0.311**	–
9. Anxiety symptoms ^c^	0.092**	-0.075**	− 0.017	0.004	0.369**	0.337**	0.330**	0.506**

Zero-order correlations are presented by Pearson correlations for continuous variables, point-biserial correlations for one dichotomous and one continuous variable, Spearman correlations for one ordinal and dichotomous or continuous variable, and chi-square tests for dichotomous variables

* p ≤ .05; ** p ≤ .01; *** p ≤ .001

^a^dichotomous variable, ^b^ordinal variable, ^c^continuous variable

#### Multiple Linear Regression Analyses- ADHD Symptoms

Collinearity statistics indicated that multicollinearity was not a concern (VIF 1.00–2.78, tolerance 0.36–0.99). Female gender (ß = − 0.16, *p* = < 0.001) was found to be significantly negatively associated with total ADHD symptoms. Both age (ß = − 0.09, *p* = 0.012) and the interaction of age and gender (ß = 0.01, *p* = 0.859) were not significantly associated with total ADHD symptoms. Further, medium (ß = − 0.16, *p* < 0.001) and high (ß = − 0.24, *p* < 0.001) parental education were significantly negatively associated with total ADHD symptoms. A sizeable proportion of variance of total ADHD symptoms (5.8%; adjusted *R*^*2*^ = 0.058, *F*[6,1319] = 14.59, *p* < 0.001) was accounted for by the predictor variables corresponding to a small effect. Among the factors associated with inattentive ADHD symptoms, female gender (ß = − 0.19, *p* < 0.001) was significantly negatively associated. Both age (ß = − 0.04, *p* = 0.339) and the interaction of age and gender (ß = − 0.04, *p* = 0.258) were not significantly associated with inattentive ADHD symptoms. Medium (ß = − 0.18, *p* < 0.001) and high (ß = − 0.25, *p* < 0.001) parental education were negatively associated with inattentive ADHD symptoms. Predictor variables accounted for 5.9% (adjusted *R*^*2*^ = 0.059, *F*[6,1319] = 14.90, *p* < 0.001) of the variance of inattentive ADHD symptoms. For hyperactive/impulsive ADHD symptoms, a significant negative association with both female gender (ß = − 0.11, *p* < 0.001) and age (ß = − 0.14, *p* < 0.001) was found. The interaction of age and gender (ß = 0.06, *p* = 0.123) was not significantly associated with hyperactive/impulsive ADHD symptoms. Medium (ß = − 0.19, *p* < 0.001) and high (ß = − 0.26, *p* < 0.001) parental education were also negatively associated with hyperactive/impulsive ADHD symptoms. Predictor variables accounted for 4.1% (adjusted *R*^*2*^ = 0.041, *F*[6,1319] = 10.50, *p* < 0.001) of the variance of hyperactive/impulsive ADHD symptoms. Full results of the multiple linear regression analyses can be found in Table 4.

**Table 4:** Results of the multiple linear regression analyses; factors predicting ADHD symptoms

Outcome variable	Total ADHD symptoms			Inattentive ADHD symptoms			Hyperactive/Impulsive ADHD symptoms		
	*b*	ß	*p*	*b*	ß	*p*	*b*	ß	*p*
Constant	0.703		< 0.001	0.935		< 0.001	0.513		< 0.001
Predictor variables									
Gender (female)	− 0.125	− 0.163	< 0.001	− 0.182	− 0.185	< 0.001	− 0.079	− 0.106	< 0.001
Age (centered)	− 0.014	− 0.092	0.012	− 0.007	− 0.035	0.339	− 0.020	− 0.135	< 0.001
age × gender	0.001	0.007	0.859	− 0.012	− 0.041	0.258	0.012	0.057	0.123
Parental education (reference: low)									
Medium	− 0.160	− 0.202	< 0.001	− 0.179	− 0.176	< 0.001	− 0.146	− 0.191	< 0.001
High	− 0.239	− 0.278	< 0.001	− 0.275	− 0.250	< 0.001	− 0.212	− 0.255	< 0.001
Migration background	− 0.071	− 0.043	0.108	− 0.149	− 0.071	0.008	− 0.010	−0.006	0.814
Adjusted *R*^*2*^		0.058			0.059			0.041	

*n* = 1326

#### Multiple Linear Regression Analyses- ADHD and Co-occurring Depression and Anxiety Symptoms

The results of the multiple linear regression analyses are presented in Table 5. Collinearity statistics indicated no multicollinearity (VIF 1.01–2.86, tolerance 0.35–0.99). Both female gender (ß = 0.08, *p* = 0.002) and age (ß = 0.07, *p* = 0.009) were found to be significantly positively associated with depression symptoms. ADHD symptoms (ß = 0.39, *p* < 0.001) were significantly positively associated with depression symptoms. Meanwhile medium (ß = − 0.12, *p* = 0.007) and high (ß = − 0.09, *p* = 0.041) parental education were significantly negatively associated with depression symptoms. A significant proportion of variance of depression symptoms (15.8%; adjusted *R*^*2*^ = 0.158, *F*[6,1319] = 42.32, *p* < 0.001) was accounted for by the predictor variables and ADHD symptoms significantly contributed to the prediction. Among the factors associated with anxiety symptoms, female gender (ß = 0.15, *p* < 0.001) was significantly associated. ADHD symptoms (ß = 0.39, *p* < 0.001) were significantly positively associated with anxiety symptoms. Medium parental education (ß = − 0.12, *p* = 0.006) was negatively associated with anxiety. Predictor variables accounted for 16.3% (adjusted *R*^*2*^ = 0.163, *F*[6,1319] = 42.94, *p* < 0.001) of the variance of anxiety symptoms, with ADHD symptoms contributing significantly.

**Table 5:** Results of the multiple linear regression analyses; factors predicting depression and anxiety symptoms in children and adolescents

Outcome variable	Depression symptoms			Anxiety symptoms		
	*b*	ß	*p*	*b*	ß	*p*
Constant	6.297		< 0.001	8.782		< 0.001
Predictor variables						
Gender (female)	0.981	0.081	0.002	2.866	0.154	< 0.001
Age (centered)	0.157	0.066	0.009	− 0.137	− 0.038	0.137
Parental education (reference: low)						
Medium	− 1.440	− 0.115	0.007	− 2.225	− 0.116	0.006
High	− 1.180	− 0.087	0.041	− 1.350	− 0.065	0.127
Migration background	0.379	0.015	0.564	0.080	0.002	0.936
ADHD symptoms	6.194	0.388	< 0.001	9.324	0.385	< 0.001
Adjusted *R*^*2*^		0.158			0.163	

*n* = 1326

## Discussion

The present study aimed to identify gender and age differences in the manifestation of ADHD symptoms, as well as illuminate associations between ADHD and co-occurring depression symptoms and anxiety among children and adolescents in the BELLA study. As the BELLA study is a population-based sample and participants were not assessed in a clinical setting, these analyses examine the degree of ADHD symptoms using validated, diagnostic instruments instead of clinical ADHD diagnoses. Findings from these analyses indicate overall low ADHD symptom severity and that girls showed significantly lower ADHD symptom scores than boys across all scales of the FBB-ADHS. Older children demonstrated lower ADHD symptom scores on the Total Score and subscale Hyperactivity/Impulsivity. Total symptoms and hyperactive/impulsive symptoms were significantly associated with age, while for inattentive symptoms no significant association was found. No significant interaction of age and gender was found for any of the ADHD scales. Both depression symptoms and anxiety were found to be associated with ADHD symptoms. Being female and older also were associated with depression symptoms. Anxiety symptoms were associated with female gender.

The changes in mean sum scores could indicate a symptom shift throughout childhood into adolescence that has been suggested elsewhere, whereby hyperactive/impulsive symptoms lessen over time and inattentive symptoms become more dominant [3, 12]. However, the small effect sizes for gender differences in the total ADHD and hyperactive/impulsive ADHD scores should be interpreted with caution and could be due to the fact that this is a population sample. These differences might be more pronounced in a clinical ADHD sample.

The significant association with gender and age and the small effect size for the Total Score of the FBB-ADHS (adjusted *R*² = 0.058) indicate that boys and younger children show higher degrees of ADHD symptoms for the Total Score than girls and older adolescents. Significant associations were also found for gender on the Inattention and Hyperactivity/Impulsivity subscales, with the subscale for Inattention having a small-medium effect size (adjusted *R*² = 0.031). This could be linked to the role that gender bias may have in parent reporting of ADHD symptoms as has been found in other research [17], [19], [18].

The relatively high scores of inattentive symptoms across gender and all age groups could support other research that has found that some children and adolescents may struggle with inattentive symptoms of ADHD, but not meet other requirements for a clinical diagnosis and thus be overlooked [40, 16]. However, our findings did not corroborate those of Willcutt et al. (2012), which suggested that a larger proportion of girls present with inattentive-type ADHD. Further, a significant interaction of age and gender with ADHD symptoms as suggested by Franke et al. [21] was not found in our study. In their meta-analysis, Willcutt et al. (2012) found that the estimated prevalence of ADHD hyperactive-impulsive type was highest in preschool children, but then declined steadily over the course of childhood into adolescence. The present study also identified this trend of reduced hyperactive-impulsive symptoms with increasing age. These findings suggest a potential evolution of ADHD symptoms, wherein hyperactive and impulsive symptoms decline with age. Further, they are in line with other studies demonstrating that depression is associated with ADHD symptoms ([23, 25, 41]) and anxiety [40], [42], [23].

This work builds upon existing research which points to gender disparities in both ADHD diagnosis and treatment. The analyses conducted here provide a foundation for other studies which could be conducted in clinical populations, providing a more in-depth perspective into ADHD and its comorbid disorders. Limitations of this study include the use of only parent reports via rating scales and structured telephone interviews. Neither diagnostic interviews nor teacher information were included in this study, nor were children and adolescents asked themselves to evaluate their own mental health. For these reasons, the data might not show the most accurate representation of the children’s and adolescents’ psychological state. Additionally, ADHD symptom severity as measured by the FBB-ADHS can be impacted by ADHD medication, a factor that was not controlled for in these analyses.

Going forward more emphasis should be put on continuing education and adhering to universal standards for clinicians working with ADHD patients as research has shown that therapists do not adhere strictly to diagnostic manuals and gender may bias their assessments [43]. As depression and anxiety are known comorbid disorders with an ADHD diagnosis [42, 44, 15], future studies could examine potential mediators of the association between ADHD and depression, since to date only few studies have explored this phenomenon [25]. Several studies have demonstrated that there is a large overlap between ADHD and stress, anxiety, and depression symptoms as age increases [40, 45], further highlighting the need to examine the ways in which these disorders interact and potentially mask one another. This is of particular importance as Schmidt et al. [46] found that ADHD-affected persons show much more severe psychological impairments than those without ADHD.

Although evidence-based pharmaceutical and behavioral treatments can ameliorate some ADHD symptoms, to date there is no “cure” or even a superior treatment approach. However, a meta-analysis by Vysniauske et al. ([47]) found that exercise had a modest positive effect on ADHD functional outcomes and a recent meta-analysis by Shephard et al. ([48]) demonstrated promising evidence that early interventions in self-regulation and other executive function deficits could help reduce ADHD symptoms.

In order to better meet girls’ unique ADHD mental health needs, health care providers, parents, and teachers need to be sensitized to and educated about the distinct ways in which ADHD manifests in girls. Namely, that the hyperactive symptoms most associated with the disorder are not always present in girls with ADHD. By this same logic, trans, non-binary, and other gender non-conforming children and adolescents struggling with ADHD might also remain unnoticed and undiagnosed if their symptoms do not align with the classic conceptions of ADHD. Therefore, more emphasis could be put on educating parents and teachers in identifying the quiet, subtle symptoms of ADHD: lack of organization, concentration problems, “dreaminess”, etc. and creating structures to better support children and adolescents in developing these skills. Continuing education regarding gender differences in ADHD presentation and the struggles of patients who mostly present the “milder” internalizing symptoms for health care providers and therapists could be also expanded. While ADHD is a sometimes contentious diagnosis, efforts need to be made to ensure that those suffering have access to and receive the care and support that they need to thrive.

## Summary

Though attention-deficit/hyperactivity disorder (ADHD) is one of the most diagnosed neurodevelopmental disorders of childhood, only few current studies address ADHD gender and age differences. The present study dimensionally evaluated gender and age differences in ADHD symptoms, as measured by a DSM-5-based parent rating scale, in 1,326 children and adolescents who participated in the two-year follow-up of the community-based BELLA study. Associations between ADHD symptoms and depression symptoms and anxiety symptoms were also examined. Multiple linear regressions revealed significant negative associations between female gender and total, inattentive, and hyperactive/impulsive ADHD symptoms. Age was found to be only significantly associated with hyperactive/impulsive symptoms. Further significant positive associations were found between ADHD symptoms and depression and anxiety symptoms. As predicted, female gender was positively associated with both depression and anxiety symptoms. These findings have the potential to inform the development of gender-specific approaches to ADHD diagnosis and treatment. Future research could more deeply examine the intersections of ADHD and depression and anxiety symptoms in children and adolescents.

## Data Availability

The data that support the findings of this study are available from the corresponding author upon reasonable request.
